# Performance-linked visual feedback slows response times during a sustained attention task

**DOI:** 10.1186/s41235-023-00487-w

**Published:** 2023-05-29

**Authors:** Ashley C. Steinkrauss, Anjum F. Shaikh, Erin O’Brien Powers, Jeff Moher

**Affiliations:** 1 Department of Psychology, Connecticut College, 270 Mohegan Avenue, New London, CT 06320 USA; 2 Department of Psychology, Pennsylvania State University, 140 Moore Building, University Park, PA 16802 USA

**Keywords:** Sustained attention, Continuous performance task, Feedback, Real-time tracking

## Abstract

In the present study, we tested a visual feedback triggering system based on real-time tracking of response time (RT) in a sustained attention task. In our task, at certain points, brief visual feedback epochs were presented without interrupting the task itself. When these feedback epochs were performance-linked—meaning that they were triggered because participants were responding more quickly than usual—RTs were slowed after the presentation of feedback. However, visual feedback epochs displayed at predetermined times that were independent of participants’ performance did not slow RTs. Results from a second experiment support the idea that this is not simply a return to baseline that would have occurred had the feedback not been presented, but instead suggest that the feedback itself was effective in altering participants’ responses. In a third experiment, we replicated this result across with both written word feedback and visual symbolic feedback, as well as in cases where the participant was explicitly told that the feedback was linked to their performance. All together, these data provide insight into potential mechanisms for detecting and disrupting lapses in sustained attention without interrupting a continuous task.

**Supplementary Information:**

The online version contains supplementary material available at 10.1186/s41235-023-00487-w.

The experience of driving a car and having a passenger exclaim “Watch out!” to alert you to a potential hazard is a reality that is occasionally experienced by even the most competent drivers. This phenomenon exemplifies how easily our minds can wander, especially when trying to balance tasks such as talking and driving, but also how a timely alert can immediately snap us back into focus. In attention-demanding tasks, lapses in focus during key moments can have dangerous consequences. Studies conducted with pilots, for example, demonstrate that repetitive tasks over an extended period of time are vulnerable to lapses in attention even when performed by experts (Casner & Schooler, 2015; Dehais et al., 2014). Losing focus as a radar operator or a radiologist might be the difference between life and death for airplane passengers and patients. Therefore, determining why, when, and how these attentional decrements, or lapses in focus, occur is crucial to understanding how we can possibly intervene to prevent hazardous and costly mistakes in tasks that require sustained attention.

## Methods of studying sustained attention

The study of sustained attention gained prominence after the experiences of radar operators in World War II. During the war, the Royal Air Force realized that radar operators were becoming less efficient over the course of a shift. In 1948, Dr. Norman Mackworth devised the Mackworth clock test, which is considered to be the first task measuring sustained attention. In this task, participants were instructed to focus on a blank clock with a black pointer (clock hand) moving in short, constant increments for 2 h, and respond whenever the clock hand moved at double the length of the short increment. This study found that attentional decrements arise after 30 min of executing a sustained attention task (Mackworth, 1948).

As technology has improved, sustained attention has most consistently been examined through tasks with a constant stream of stimuli in which the participant continuously responds to targets, thus allowing for a more fine-grained analysis of behavior. Two types of tasks have emerged as standard measures of sustained attention—CPTs and Not-X-CPTs. CPT stands for the Continuous Performance Task. In this task, participants are presented with one letter (e.g. “A”) during each trial. They are instructed to only perform a keypress when the target letter, often the letter “X,” appears on the screen (e.g., Cohen, 2011). Not-X-CPTs, also known as the Conners Continuous Performance Task, similarly present a stream of letter stimuli sequentially. However, for these tasks, participants are directed to perform a keypress when every letter except the letter “X” appears on the screen—these can be referred to as “no-go” trials (Conners & Sitarenios, 2011). CPTs are generally longer tasks and have been shown to be a reliable measure of vigilance decrements, or periods of declined accuracy and performance due to lapses of sustained attention (Riccio et al., 2002). On the other hand, Not-X-CPTs have been inconsistent in measuring vigilance decrements, but studies have demonstrated that they are a dependable measure of sustained attention based on variability in response times (RT) (Folsom & Levin, 2013). In these tasks, vigilance decrements are often noted by a decline in accuracy or performance. More specifically, not-X-CPTs utilize faster RT responses to predict errors committed on “X” trials due to vigilance decrements (Cheyne et al., 2006; Rosenberg et al., 2013).

Robertson’s Sustained Attention to Response Task (SART) pioneered the go/no-go continuous performance task (CPT). In the SART, participants are instructed to perform keypresses for a random, sequential stream of numbers and withhold key presses to the number three (the no-go target) that appears infrequently. The SART was developed to study vigilance decrements in patients who had experienced traumatic brain injuries, but has since been utilized in numerous studies on sustained attention. The SART demonstrated that erroneous keypresses on go trials (non-target stimuli) could be predicted by a decrease in RT directly preceding the error (e.g., Robertson et al., 1997). Despite the fact that the SART is a CPT, this study demonstrated that the SART has some of the benefits of not-X-CPTs and paved the way for future iterations of CPTs that can reliably measure vigilance decrements and predict lapses of sustained attention based on variability in response time.

In an effort to fully examine both of these measures of sustained attention simultaneously, Rosenberg et al. (2013) created a new task called the gradual-onset continuous performance task (gradCPT), which allows for sustained attention to be measured in terms of both response times and vigilance decrements. In the gradCPT, participants are presented with images of either a rare non-target or a target on each trial and are instructed to perform a keypress for each target stimulus and withhold a response for the rare non-target stimuli. Using this novel sustained attention task, Rosenberg et al. (2013) found that increased variability in RT resulted in more errors of commission, or erroneous responses to non-target stimuli. Furthermore, commission errors steadily increased over the course of the task. This task has been subsequently used to study many aspects of sustained attention such as brain networks associated with fluctuations in sustained attention (Esterman et al., 2012), sustained attention over the lifespan (Fortenbaugh et al., 2015), and the minimal impact of rewards on sustained attention (Esterman et al., 2014).

## Theories and approaches to improve sustained attention

In order to target lapses in sustained attention for intervention, it is necessary to understand why they occur. There are at least two potential explanations for why lapses of sustained attention occur that have been discussed in the literature. The overload theory attributes attentional decrements in sustained attention tasks to resource depletion. The overload theory suggests that there is a limited amount of resources available for cognitive processing and that these resources deplete due to attentional exertion and increased difficulty of a task (Grier et al., 2003; Pattyn et al., 2008; Ralph et al., 2017; Smit et al., 2004; Thomson et al., 2015; Warm et al., 2008). Based on this theory, interventions such as rest breaks are the best way to improve attentional decrements in a sustained attention-demanding task. Underload theory, on the other hand, attributes attentional decrements to the lack of arousal and the monotonous nature of a task. According to underload theory, in order to increase attention, arousal must be increased such as with an alternate task that engages the attention of the participant (Manly et al., 1999; Pattyn et al., 2008; Ralph et al., 2017; Thomson et al., 2015). These two contrasting theoretical approaches suggest that different potential interventions for reducing lapses of sustained attention may be effective depending on the nature of the task.

Multiple studies have investigated ways to reduce attentional decrements. For example, one study examined the effect of live neurofeedback on the attentional state of an individual. Participants were placed in a functional magnetic resonance imaging (fMRI) scanner and were shown a composite image of a face and a scene and asked to focus on one of the two images. The attention of the participant was tracked using whole-brain fMRI analysis, specifically real-time fMRI (rtfMRI) with multivariate pattern analysis, to provide feedback. When the participant's attention began to slip as measured by rtfMRI, the target of the composite image became harder to see as the images blurred together even more. Likewise, when attention improved the composite image target became clearer and easier to decipher. Utilizing rtfMRI, the researchers discerned that live neurofeedback training affected regions of the brain including the frontal cortex, ventral temporal cortex, and basal ganglia (specifically the striatum and globus pallidus). Critically, the study found that their live neurofeedback mechanism decreased attentional decrements over time (deBettencourt et al., 2015).

Simpler behavioral approaches can also be used to improve performance on sustained attention tasks. Ralph et al. (2017) found that both taking breaks and engaging in an alternate task decreased participants’ response times, thereby improving their sustained attention. Participants in that study engaged in a mentally challenging task where they completed a version of the Mackworth clock task. In this task, participants were asked to watch a clock’s hand move. If the clock hand “skipped” a tick, then they had to click a button. Three versions of this task were administered: one where participants performed the Clock Task continually, one where participants were instructed to take a rest break, and one where participants were instructed to complete another visuospatial task (the Car Task) during a break period. Ralph et al. (2017) demonstrated that interrupting a repetitive task, even if the interruptions were demanding tasks themselves, could reduce lapses of sustained attention. In addition to exploring the effects of breaks and alternate tasks, the effects of monetary incentives on sustained attention have also been examined. Specifically, Esterman et al. (2016), found having a large looming loss (e.g. loss of money) as a consequence of a potential single error reduced the trend of increasing errors over the course of the task. Interestingly, small rewards over time improved the overall performance, but observers still experienced the same trend of increased errors over time.

The studies described above illustrate multiple ways in which sustained attention decrements can be reduced. However, many of these approaches are not easily applicable to real life situations in which sustained attention is necessary. For example, a study that examined ways to improve sustained attention in pilots found that interruptions, or having the pilot engage in other cockpit activities, were a distraction and led to more attentional decrements and more mistakes (Casner & Schooler, 2015). This example highlights the reality that breaks, alternate tasks, or looming threats do not align with many tasks that require sustained attention due to the fact that taking a break or changing one’s attentional target could result in major mistakes. Furthermore, in many real-world tasks these reduction strategies would not be feasible. For example, tasks such as operating heavy machinery or conducting surgery may require hours of continuous sustained attention without the opportunity for breaks. Due to this reality, the aim of the current study is to examine more applicable alternatives in which attentional decrements may be attenuated with simple, real-time interventions that respond to changes in a participant’s behavior without interrupting the task.

## Present research with effects of alerts on attention

One potential mechanism to reduce attentional decrements involves deploying an alert when an individual is most vulnerable to lapses in focus. The use of an alert system is particularly relevant to the operation of vehicles such as cars and planes. Much of the literature employs either tactile or auditory alerts in an effort to minimize the consequences of attentional decrements and reinstate attention on the assigned task (e.g., Graham, 1999; Hester et al., 2017; Lees et al., 2011; Nees et al., 2016). There is, however, a relatively limited literature available on real-time visual feedback alerts improving performance in a sustained attention task.

A predominant focus of the literature on alert systems centers around improving the attention of vehicle operators through simulations in which alerts are employed. For example, Fitch et al. (2007) found that seat vibration alerts in a car, paired with the location on the seat representative of the region of the car where a crash was going to occur, were the most effective at alerting a driver to a potential crash. This study suggests that interventions from alerts right before a potential mistake can help participants maintain accuracy. Gonzalez et al. (2012) also examined alerts while driving, finding that while auditory alerts increase urgency in driving, they also increased annoyance. Wiese and Lee (2001) also looked at auditory alerts on driver performance and attitude and similarly found that annoyance was a significant characteristic that can affect workload and performance. Given these findings, these results suggest that auditory alerts may have unintended negative consequences, especially on the participants’ opinions of alerts.

Returning to the discussion on sustained attention, deBettencourt et al. (2019) examined the relationship between sustained attention and working memory using real-time tracking of sustained attention based on the response times of the participants. Participants were instructed to press keys based on the shapes of a visual array. To track sustained attention in real time, deBettencourt et al. monitored participants’ cumulative RT and standard deviation as well as the trailing mean of the last three trials prior to each trial. Whenever this trailing mean was less than the cumulative mean minus the cumulative standard deviation or greater than the cumulative mean and cumulative standard deviation combined, a working memory task probe was deployed that probed for the colors of all shapes during the previous trial. When there was low attention as reflected by RTs that were shorter than one standard deviation below the mean, participants remembered fewer items from prior trials (deBettencourt et al., 2019). This indicates that lapses in attention, indicated by fast response times, lead to worse working memory performance.

This novel probing method is a promising approach to studying fluctuations in attention and implementing changes in real-time, without interrupting the task at hand—a feature of vital importance to occupations where taking a break or completing alternate assignments during sustained attention tasks is not feasible and may even cause harm.

In the present study, we adapted the approach from deBettencourt et al. (2019) to test how the appearance of visual feedback, prompted by user performance, can change behavior in a sustained attention task. We used real-time tracking of sustained attention (similar to deBettencourt et al., 2019) to detect when participants were likely to be in a less focused attentional state, and feedback epochs were presented when attention appeared to be declining based on the live-tracking of RT. Feedback consisted of words (“Correct!” or “Incorrect”) appearing under the image or colored circles surrounding the image for short bursts of 5 trials according to how the participant responded. Importantly, this intervention did not require any pausing of the task.

In Experiment 1, participants completed a version of the gradCPT (adapted from Fortenbaugh et al., 2015). Short bursts of feedback were presented either when participants were in a less-focused state based on our real-time tracking of RT, or at predetermined times. This allowed us to determine whether changes in performance following feedback epochs occurred only because of the feedback itself, or because the feedback was triggered by specific patterns in participant performance. In Experiment 2, all participants were subject to the real-time RT-based feedback. For each period of shorter-than-usual RT that was detected, feedback epochs were either displayed to the participant (“visible” feedback) or hidden from the participant (“invisible” feedback), allowing us to determine whether changes in performance observed in Experiment 1 were due to the feedback itself, or to a return to baseline that naturally occurs over time even if feedback was not presented. Across both experiments, we hypothesized that when feedback was triggered by periods of shorter-than-usual RT, commission errors would be attenuated and RTs would increase in trials immediately following the feedback, reflecting a return to a more focused attentional state. In other words, we predicted that the feedback epochs would serve as a useful alert that improved participant performance and forestalled potential upcoming errors. Finally, in Experiment 3, we manipulated the type of feedback, using either written words as in Experiments 1 and 2, or visual symbols in the form of green and blue circles. We also manipulated the participants’ knowledge of the connection between the feedback and their own performance. This allowed us to explore the extent to which performance-triggered feedback might increase RT across a broad variety of contexts.

## Experiment 1

### Methods

#### Participants

101 participants completed the experiment (females = 51 and males = 47; mean age = 28 years; three participants failed to report demographic information); we removed from analyses all participants who went at least twenty trials without pressing a key, under the assumption that this would include participants who were not engaged with the task throughout the experiment. This resulted in the elimination of three participants, leaving 98 total participants. All participants had normal or corrected-to-normal vision. Experiments were posted and publicly available on Prolific (http://www.prolific.co). Participants were required to have a United States-based location. All participants provided informed consent and were provided monetary compensation for their participation. The protocol was approved by the Connecticut College Institutional Review Board. Sample size was based on prior studies using a similar task (deBettencourt et al., 2019; Rosenberg et al., 2013) but with larger samples to compensate for potentially more variable data from online data collection as resources would allow.

#### Stimuli

**Fig. 1:**
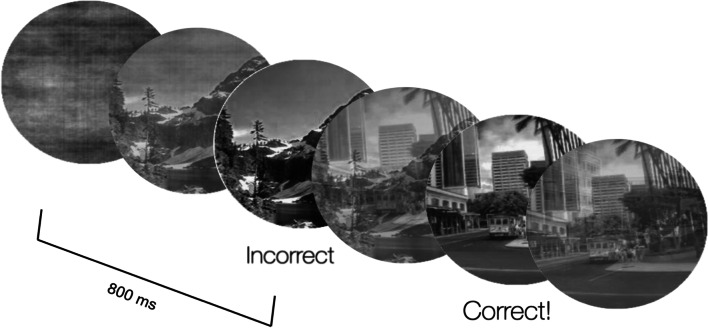
Examples of feedback given to participants. *Note:* Each image faded in until appearing at full saturation after 800 ms, then faded out as the next image began appearing. The first feedback trial includes a mountain scene in which the participant incorrectly pressed the “M” key on the trial (commission error) and the second feedback trial includes a city scene in which the participant correctly hit the “M” key on the trial (correct commission)

We used a version of the gradCPT adapted from Fortenbaugh et al. (2015). Participants were shown a series of sequential images that were either cities (85% of the time) or mountains (15% of the time). Note that we used a higher proportion of mountains relative to prior studies in order to increase the number of no-go trials during critical periods of interest. There were 10 round grayscale images measuring 200 × 200 pixels from each category that were used. Images were presented in a randomized order with the exception that an image was never presented on consecutive trials, and images continuously faded in for 800 ms from a starting point of 0% opacity until they reached 100% opacity, then faded out for 800 ms in the opposite direction of opacity. Each segment of the experiment began and ended with a gray circle of the same dimensions. Images overlapped so as one image was fading in, the next image was fading out at the same time, all at the same central location. When feedback was presented it appeared in the form of text below each image that read either “Correct!” or “Incorrect.” These messages stayed on the display for 500 ms (see Fig. 1 for examples of the feedback). Feedback was triggered either by a response, or by the end of the trial if no response occurred during the trial. During the primary task, when feedback was presented it was shown for epochs of 5 consecutive trials. We will subsequently refer to these periods as *feedback epochs.* The timing of the appearance of these feedback epochs was determined as a function of experimental condition (see procedure for more details).

We used custom JavaScript code for stimulus presentation adapted from PsiTurk (Gureckis et al., 2016). At the end of the experiment, we asked participants a series of questions from an ADHD self-report questionnaire (Adult ADHD Self-Report Scale [ASRS-v1.1] Part A) along with a simple self-report mind-wandering questionnaire (see “Appendix A”) and a series of questions about their awareness and feelings about the feedback epochs. The latter included questions about whether the participant thought the feedback epochs were responding to their performance, whether they found the feedback epochs helpful, whether they thought the feedback epochs had a positive impact on their performance, and whether they thought the feedback epochs were annoying. ASRS data were collected for a separate project but are publicly available along with other subject data at our OSF website (https://osf.io/mkgej/).

#### Procedure

Participants were told they would see a series of pictures of cities and mountain scenes and every time they saw a city scene they should press the ‘M’ key, but they should withhold from pressing the “M” key when they saw a mountain scene. Participants completed 50 practice trials that ramped up in speed and difficulty until they were practicing the primary task. All practice trials had city and mountain scenes appear equally, unlike the experiment where mountains appeared on a randomly selected 15% of all trials. Practice trials were broken apart into three sections. The first section had no transitions, slow speed, and feedback (10 trials). The second section had transitioning images, slow speed, and feedback (20 trials). The third section had transitioning images, full pace, and no feedback (20 trials). Participants saw instructions before each set of practice trials informing them of the changes. For each screen they were instructed to be as accurate as possible. After practice, participants completed 4 blocks of 100 trials each with no breaks in between.

There were two between-subjects experimental conditions regarding feedback during the primary task. Participants were randomly assigned to one of these two groups prior to the experiment. For both groups, no feedback was presented during the first block of trials in order to establish a stable mean RT for each participant. For the Predetermined group, participants were randomly assigned to see either 3, 4, 5, or 6 feedback epochs. Each epoch began at a predetermined trial number. The spacing between each epoch was roughly equal, with shorter spacings for participants who received more frequent feedback epochs; however, the exact trial on which each epoch appeared was randomly jittered for each participant by up to 5 trials in either direction to make the appearance of the epochs less predictable. For the Triggered group, feedback epochs were triggered by a period of atypically rapid responding. These periods were defined as three consecutive city trials with correct responses in which the participant’s mean RT was at least 1 standard deviation lower than their cumulative mean RT up to that point of the experiment (similar to deBettencourt et al., 2019). After one feedback epoch was triggered, the next epoch could not be triggered until at least 30 additional trials had passed. Pressing the key was considered a *correct commission* on city trials and a *commission error* on mountain trials.

#### Data analysis

Keypresses were linked to trials using the same algorithm as Fortenbaugh et al. (2015) in which a keypress was assigned to a trial using an iterative procedure taking into account when the most recent keypress was made, the relative time at which recent images appeared, the trial type of the most recent and current trial, and whether a response was already recorded for the prior trial. Once assigned, RT was considered relative to the onset of an image, such that a response time of less than 800 ms would be a keypress that occurred before the image reached 100% opacity.

### Results

#### Self-report

We conducted independent-samples t-tests between the predetermined and triggered groups to determine whether there were group-level differences on self-report questions (see “Appendix” for question details). A small number of participants did not respond to all questions—for each analysis, we included all participants who answered the particular questions in that analysis. The groups showed no difference in the extent to which they thought the feedback epochs were responding to their performance, *t*(86) < 1. Critically, this suggests that participants were not aware of when the feedback was being triggered by their performance as opposed to when feedback epochs were presented at predetermined times that were entirely independent of their performance. In other words, participants were not consciously aware of the key manipulation of the present study. We also found no differences across other self-report questions related to mind-wandering or participants’ perceptions of the feedback epochs, all *t*s < 1. On average, participants found the feedback epochs to be annoying (M: 3.83) more so than helpful (M: 3.11) or positively affecting their performance (M: 3.49), though none of these comparisons reached statistical significance, *p*s > 0.05. See Table 1 for all means and test statistics.

**Table 1:** Statistic descriptives using t-test for equality of means

	Predetermined	Triggered	*t*-statistic
	Mean (SD)	Mean (SD)	
Helpful	3.16 (2.01)	3.07 (2.30)	0.406
Changes	3.00 (1.99)	3.07 (2.12)	− 0.051
Positive	3.65 (2.05)	3.33 (2.07)	0.921
Annoying	3.65 (2.19)	4.00 (2.48)	− 0.835
Mind-wandering	3.28 (1.65)	3.23 (1.72)	0.142

*p* ’s > .05

#### Total feedback epochs

Our goal in the present study was to approximately match the total number of feedback epochs across our two groups. However, because it was not possible to predict exactly how many feedback epochs would occur in the triggered group, this was an approximation done before data collection. Overall, more feedback epochs did occur in the triggered group (M: 5.5) compared to the predetermined group (M: 4.7), *t*(76.50) = -3.89, *p* < 0.001.

There were two primary approaches we used to determine the extent to which feedback impacted performance. The first is to examine overall group level differences on primary metrics of performance we refer to these as global analyses. The logic for these analyses is that perhaps performance-triggered feedback leads to global changes in performance relative to feedback that is not related to an individual’s performance. The second approach was to examine local changes related to feedback epochs—that is, are there overall or group-level changes that are caused in the immediate aftermath of the sudden appearance of visual feedback. We refer to these as local analyses.

#### Global analyses

We examined measures of response time (RT), coefficient of variation (CV, calculated as the SD divided by the mean for a given condition, multiplied by 100), commission errors, and correct commission rates across each block of trials. Responses on trials during which feedback was occurring were not included in these analyses. Finally, we considered the possibility that group level differences took time to emerge over the course of the experiment; thus, we looked at each of these measures only within the final block of 100 trials in a separate analysis.

**Fig. 2:**
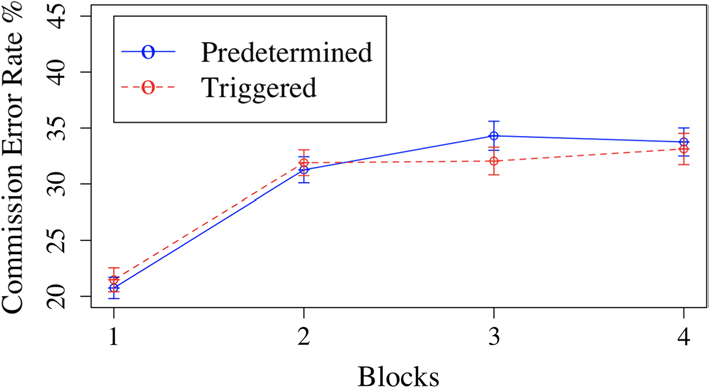
Commission error rates for predetermined and triggered conditions by block. *Note:* Blocks were evenly separated across trials so Block 1 is the one-fourth of the given experiment trials

A 2 × 4 ANOVA was run on RT, CV,^1^ commission errors, and correct commission rates with factors of block (1, 2, 3, or 4) and condition (triggered or predetermined). For RT, there were no main effects of condition *F*(1,96) = 0.30, *p* = 0.58 or block *F*(3,288) = 1.5, *p* = 0.21, nor an interaction *F*(3,288) = 0.04, *p* = 0.99. For CV, there was a significant effect of block *F*(3,262) = 34.76, *p* < 0.001, η_p_^2^ = 0.27 but no effect of condition *F*(1,96) = 0.06, *p* = 0.81, nor an interaction *F*(3,262) = 0.64, *p* = 0.57. The main effect of block reflects an increasing CV as the experiment progressed. For commission errors, there was no effect of condition, *F*(1,96) = 0.02, *p* = 0.88, nor an interaction, *F*(3,288) = 0.30, *p* = 0.82. There was a significant effect of block, *F*(3,288) = 20.92, *p* < 0.001, η_p_^2^ = 0.18 (Fig. 2). These follow similar patterns to previous findings (e.g., Rosenberg et al., 2013) in which errors increase in the gradCPT as the task progresses. For correct commission rates, there was again no main effect of condition nor an interaction, *p*s > 0.05. There was again a significant effect of block, *F*(3,288) = 13.38, *p* < 0.001, η_p_^2^ = 0.12, with a similar pattern to correct commission rates, where performance decreased as time-on-task increases. See Additional file 1: Table S1 for means and standard deviations. Finally, we conducted independent samples t-tests to compare commission errors, correct commission rates, CV, and RT for just the last 100 trials across the two different conditions; no results were significant, all *p*s > 0.05.

All together, we saw no evidence of a global change in performance generated by the presence of feedback epochs. This does not mean that performance-triggered feedback could not lead to overall improvements in performance; rather, it suggests that the current approach does not do so, either because the impact of feedback epochs is the same regardless of whether the feedback is triggered by performance or not, or because the feedback does not improve performance in this context. Future studies should directly compare these conditions against a condition in which no feedback is presented.

#### Local analyses

To better understand the impact of feedback epochs on performance, we examined RT, CV, correct commission rates, and commission error rates immediately before and after feedback epochs across both conditions. Because commission error rates can only be measured when mountain trials appear, and those only appear in 15% of all trials, we chose to examine means from windows of the 8 trials immediately prior to the onset of feedback epochs and the 8 trials immediately following the conclusion of feedback epochs; all but one participant had at least one trial in each condition for this analysis (that participant was removed for these analyses). A 2 × 2 mixed factorial ANOVA was run with factors of condition (predetermined vs. triggered) and time (before vs. after a feedback epoch) for each dependent variable.

**Fig. 3:**
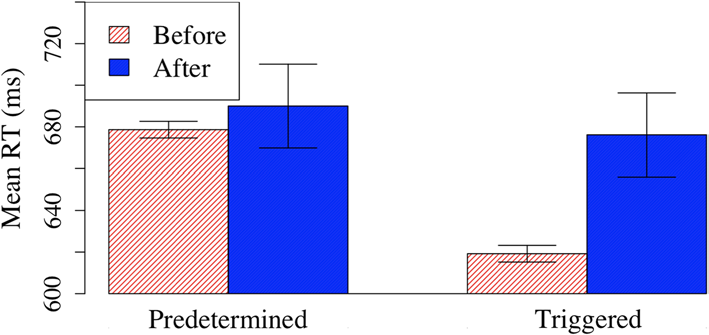
Mean RT of before and after feedback epochs for predetermined and triggered conditions

For RT, there were main effects of condition *F*(1,96) = 6.36, *p* = 0.013, η_p_^2^ = 0.06 and time *F*(1,96) = 26.16, *p* < 0.001, η_p_^2^ = 0.21 that were mediated by a significant interaction, *F*(1,96) = 11.68, *p* = 0.001, ηp^2^ = 0.11 (Fig. 3). Follow-up t-tests showed that RT increased following feedback epochs in the triggered condition (before: 619 ms, after: 676 ms), *t*(47) = 6.66, *p* < 0.001). However in the predetermined condition, the difference in RT failed to reach significance (before: 679 ms, after: 690 ms), *t*(49) = 1.11, *p* = 0.27). These results indicate that triggered feedback linked to performance did alter participants’ behavior in a way that feedback independent of performance did not. Specifically, the triggered feedback significantly slowed participants’ responses.

There was a main effect of time on all other measures as well. CV was higher following feedback epochs (before: 19, ms; after: 22 ms), *F*(1,96) = 10.44, *p* = 0.002, η_p_^2^ = 0.10. Commission error rates were higher following feedback epochs, (before: 26.2%, ms; after: 36.4% ms), *F*(1,94) = 10.10, *p* = 0.002, η_p_^2^ = 0.10. For the commission error rate analysis, two participants had no mountain trials in at least one of the conditions, and thus were removed from that analysis. And finally, correct commission rates were lower following feedback epochs, (before: 96.6%, ms; after: 95.5%, ms), *F*(1,96) = 4.51, *p* = 0.04, η_p_^2^ = 0.05. This suggests that feedback epochs on the whole were quite disruptive; rather than improving performance by re-focusing on the task, these feedback epochs appeared to distract participants and disrupt focused attention.

There was a significant main effect of condition on commission error rates, *F*(1,94) = 4.13, *p* = 0.045, η_p_^2^ = 0.04, with higher commission error rates in the predetermined condition (35.1%) compared to the triggered condition (27.5%). However, this difference was not observed when the global data were analyzed as reported in the earlier section, and may be an artifact of differences in the pre-feedback requirements in the triggered condition (e.g., a series of consecutive accurate responses that were required to trigger feedback). Thus, we caution against interpreting this result as suggesting that overall commission error rates were truly lower in the triggered condition.

Critically, there were no other main effects of condition or interactions for any of these other measures, all *p*s > 0.05. In other words, these measures were otherwise not differentially affected by whether the feedback was triggered or predetermined; RT was the only measure that was differentially affected. Together, these data highlight two key findings. First, feedback epochs on the whole were disruptive rather than helpful to performance. Second, however, these disruptive feedback epochs were successful in slowing participants down when they were responding rapidly—normally a key indicator that they are losing focus and likely to soon commit an error.

**Fig. 4:**
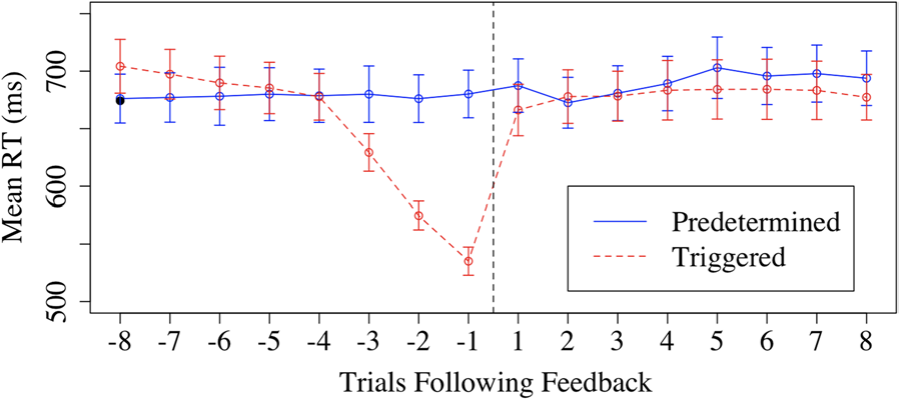
Time window of RT before and after feedback epochs for the predetermined and triggered conditions. *Note:* The negative trials on the x-axis are trials before the feedback given and the positive trials are the trials following feedback

Because the size of the time window was somewhat arbitrary, we conducted a follow-up analysis examining RT at a more fine-grained level (Fig. 4). Starting at the trial immediately before and immediately after feedback epochs, we created a moving window average of 3 trials to examine RT. For example, trial 1 would include the first trial following the conclusion of the feedback epoch, and the two trials after that. In these windows, we only include RT for a given trial in the mean for each participant if it is a city trial and the response was accurate. For trial -1, this value would include the trial immediately before the feedback epoch began, and the two trials preceding that trial. As can be seen in Fig. 4, there is a clear pattern in the triggered trials in which RT was decreasing leading up to the triggering of the feedback epochs, as would be expected based on the algorithm that was used to trigger those feedback epochs. Notably, following feedback, RT in the two conditions looks almost identical. In a three-way ANOVA with factors of time, condition, and trial number, there was a significant three-way interaction, *F*(7.96) = 26.51, *p* < 0.001. To better parse this, we conducted separate two-way ANOVAs with factors of trial number and condition for the trials before the feedback epochs and the trials after. Confirming the description above, an interaction was observed for the trials before the feedback epoch, *F*(7,672) = 55.51, *p* < 0.001, ηp^2^ = 0.37, but not after, *F*(7,672) = 0.75, *p* = 0.63.

## Experiment 2

The data from Experiment 1 suggest that feedback epochs that are triggered by periods where participants are responding faster than normal can alter subsequent behavior, as RT is subsequently slowed. Conversely, randomly appearing feedback epochs have little effect on RT. However, a possible alternative explanation is that the feedback epochs are not causally involved in the post-feedback slowing we observed in the triggered condition. That is, participants would have slowed down anyways even if we had not presented them with feedback epochs, because the periods that preceded that feedback involved atypically fast responding. Furthermore, differences in the frequency of feedback epochs across conditions potentially complicated the interpretation of analyses from Experiment 1.

To address these concerns, in Experiment 2 we generated a within-subjects comparison in which periods of atypically short RTs would only trigger feedback epochs half the time. The other half of the time, the feedback epochs were still triggered by the algorithm but made invisible, such that from the participant’s perspective, nothing changed. This way, we can directly compare what happens to a participant's pattern of responses following a period of atypically short RTs as a function of whether feedback epochs were triggered or not.

### Methods

#### Participants

48 participants completed the experiment (females = 18, males = 29, nonbinary = 1; mean age = 28); four were removed from analysis using the same criteria as Experiment 1. Because of the within-subjects design as compared to the between-subjects design of Experiment 1, we aimed to collect a sample size approximately half that of Experiment 1. All participants had normal or corrected-to-normal vision. All other aspects were identical to Experiment 1.

#### Stimuli and procedure

Experiment 2 was identical to Experiment 1 except where otherwise noted. There was no predetermined condition in Experiment 2. Instead, feedback epochs were generated for all participants based on their performance using an algorithm similar to Experiment 1. The only difference was that half the time the algorithm would have triggered a feedback epoch, the feedback was not displayed. Therefore, there were *visible* feedback epochs which were similar to Experiment 1, and *invisible* epochs in which participants would have triggered a feedback epoch, but feedback was not displayed. These appeared in alternating order, such that if the prior period of atypically short RTs triggered a visible feedback epoch, the next one would trigger an invisible feedback epoch. It was randomly assigned for each participant whether the first period of short RTs would trigger a visible or invisible feedback epoch.

### Results

#### Self-report

As in Experiment 1, we collected the same self-report measures. Here, however, there were no between-subjects variables with which to make a comparison. As in Experiment 1, scores were higher for reporting the feedback to be annoying (M: 4.70) rather than helpful (M: 3.44) or positively affecting performance (M: 3.98). Responses were significantly higher for annoying compared to helpful, *t*(39) = 2.09, *p* = 0.04, but the other comparisons were not significant, *p*s > 0.05. See Table 2 for means and standard deviations.

**Table 2:** Statistic descriptives of means and standard deviations

	Mean (SD)
Helpful	3.44 (2.26)
Changes	3.02 (2.34)
Positive	3.98 (2.17)
Annoying	4.70 (2.34)
Mind-wandering	3.42 (1.76)

#### Local analyses

**Fig. 5:**
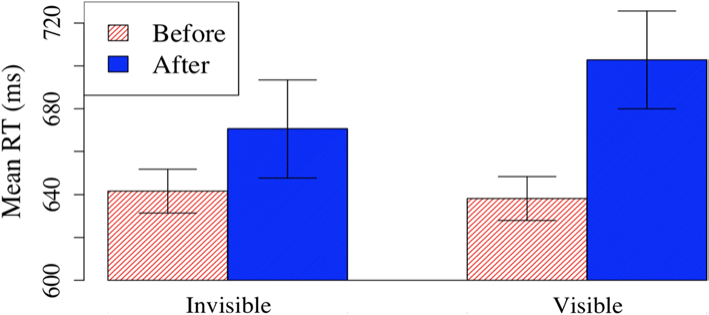
Mean RT for invisible and visible feedback before and after feedback epochs

We conducted 2 × 2 ANOVAs with factors of feedback type (visible vs. invisible) and time (before vs. after feedback epochs), using windows of 8 trials as in Experiment 1. The key analysis was focused on RT as that was the variable that was differentially affected by performance-triggered feedback epochs in Experiment 1. There were main effects of feedback type, *F*(1,43) = 4.17, *p* = 0.047, η_p_^2^ = 0.09, and time, *F*(1,43) = 38.83, *p* < 0.001, η_p_^2^ = 0.48, mediated by a significant interaction, *F*(1,43) = 5.07, *p* = 0.03, η_p_^2^ = 0.11 (Fig. 5). Follow-up t-tests revealed that RT was slower following feedback epochs in both feedback conditions, but the magnitude of the slowing was much larger in the visible condition (65 ms) compared to the invisible condition (29 ms). Furthermore, after feedback, RTs were longer in the visible condition (703 ms) compared to the invisible condition (671 ms), *t*(43) = 2.48, *p* = 0.02. In other words, the slowing that occurs following feedback epochs is not simply the result of a reversion to the mean; rather, the visible feedback itself plays a role in slowing participants down.

As in Experiment 1, more commission errors occurred after the feedback epochs (33.2%) than before (18.6%), *F*(1,33) = 9.18, *p* = 0.005, η_p_^2^ = 0.22. Ten participants were excluded from the commission error analysis because they had no mountain trials in one of the conditions; because this experiment was within-subjects, there were in some cases fewer total observations in some of the conditions relative to Experiment 1. No other main effects or interactions were significant for commission error or for correct commission rates,* p*s > 0.05.

There was a main effect of visibility for CV, *F*(1,43) = 5.82, *p* = 0.02, η_p_^2^ = 0.12, mediated by a significant interaction, *F*(1,43) = 5.13, *p* = 0.03, η_p_^2^ = 0.11. Follow-up t-tests revealed a significant decrease in CV (3 ms) following invisible feedback epochs, *t*(43) = 2.14, *p* = 0.04, but no significant change following visible feedback,* t*(43) = 1.36, *p* = 0.18. The direction of CV in the visible condition was the same as in Experiment 1 (an increase of 2 ms) despite not reaching statistical significance. The reduction in CV in the invisible feedback condition may reflect a return to baseline performance that includes reduced variability relative to the period of fast responding that triggered the feedback.

**Fig. 6:**
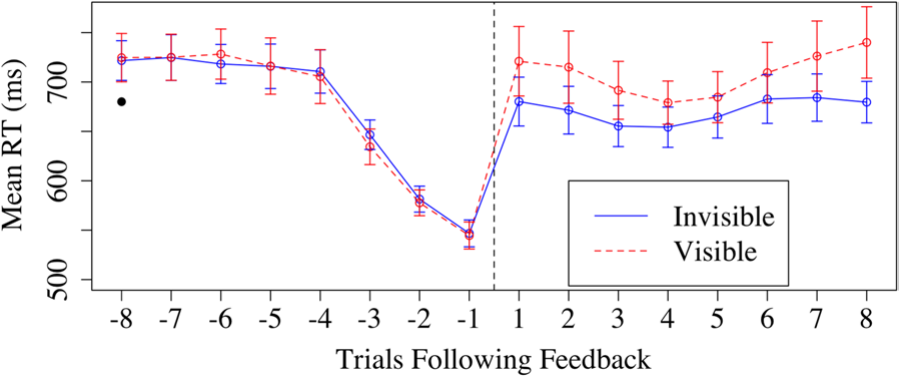
Mean RT for Trials Following Invisible and Visible Feedback Epochs. *Note:* The negative trials on the x-axis are trials before the feedback given and the positive trials are the trials following feedback. Each trial on the y-axis indicates an average three trials of a moving window

A similar analysis as in Experiment 1 was conducted examining a moving average of trials preceding and following the feedback epochs (Fig. 6). Performance on the trials before the feedback epochs were approximately equivalent across conditions. We conducted a 2 × 2 × 8 ANOVA with factors of timing, feedback type, and trial. There were main effects of feedback type, timing, and trial, *p*s < 0.05, that were mediated by interactions. Critically, there was an interaction between timing and feedback type, *F*(1,301) = 5.32, *p* = 0.03, η_p_^2^ = 0.11. Simple main effects analyses showed that there was an effect of feedback type after feedback epochs, *F*(1,43) = 7.99, *p* = 0.01, with longer RTs following visible feedback (708 ms) compared to invisible feedback (672 ms), but no effect of feedback type before feedback epochs, *F*(1,43) = 0.02, *p* = 0.89. There was also an interaction between timing and trial, *F*(7,301) = 59.74, *p* < 0.001, η_p_^2^ = 0.58, reflecting a large effect of trial on RT in the trials before feedback epochs but not after as seen in Fig. 6. No other interactions reached significance, *p*s > 0.05.

Critically, these results show that visible feedback slows RT; with invisible feedback, RT remains shorter for at least the length of time we analyzed. In other words, without intervention, participants continue responding faster than normal for a long time.

The results from Experiment 2 show promising evidence that when feedback is triggered at a particular moment in time, the feedback alters response patterns by slowing participants down. However, one issue with the approach utilized in Experiment 2 may be the distractibility of the words themselves; for example, prior research has suggested that words are automatically read regardless of task-relevance (see e.g., Augustinova & Ferrand, 2014, for a review), and this process may have taken limited resources away from the primary task in the current study. Furthermore, the presentation of words as feedback may be unfeasible in many real-world situations.^2^ In Experiment 3, given the potential distractibility of words, we introduce a new form of feedback, a colored circle surrounding the stimulus to indicate accuracy. Additionally, we explore the effects of explicit knowledge about the link between performance and feedback by introducing a condition in which we inform participants that feedback epochs are tied to their performance in the task instructions. Robison et al. (2021) examined the effects of altering task instructions on a sustained attention task, finding that participants who received specific goal-setting instructions (easy and difficult) at the outset of the experiment demonstrated reduced RTs and vigilance decrements. In their second experiment, they coupled the goal setting conditions with RT feedback at the end of each block, which was found to increase task engagement. This study suggests that explicit knowledge of the difficulty of a task given to participants in task instructions can modify performance on a sustained attention task. In Experiment 3, we wanted to explore if explicit knowledge that feedback epochs were linked to performance could improve task performance and increase task engagement. It is possible that explicit knowledge could help participants better understand that the feedback is there to help them alter their behavior during lapses of sustained attention. Alternatively, they may be upset at the idea that they are being told that their performance is declining, or they may feel increased pressure to perform following feedback that could backfire (see Belletier et al., 2015). By including these two major changes in feedback epochs and task instructions, Experiment 3 aims to build on the success of Experiment 2 and address potential flaws with the previous approach.

## Experiment 3

### Methods

#### Participants

394 participants completed the experiment and (females = 181, males = 197, nonbinary = 5; declined to respond = 11; mean age = 27.98); forty-one were removed from analysis using the same criteria as Experiment 1.

#### Stimuli and procedure

Experiment 3 was identical to Experiment 2 except where otherwise noted. Between-subjects conditions were broken up into feedback epochs (word feedback vs. visual circle) and instruction type (explicit vs. implicit). The word feedback condition was identical to the feedback epochs given in Experiment 2. In the visual circle condition, a green (correct) or blue (incorrect) circle appeared around the stimulus during feedback epochs instead of word feedback, with the same timing and triggering parameters as the word feedback.

In the explicit instruction type condition, participants were told at the beginning of the experiment that word or circle feedback epochs would appear based on their performance. Specifically, they were told: “Based on your performance, we will identify times when we think you might be prone to make mistakes. During those periods, we will give you brief periods of feedback to help you stay focused.” In the implicit condition, this instruction was replaced with the instruction, “Please do your best to stay focused.” Thus, in the explicit condition, participants were aware that feedback was tied to their performance and triggered at times when their performance indicated they were losing focus, while in the implicit condition participants were not told that the feedback was related to their performance.

In the explicit condition (181 participants included), there were two between-subjects conditions, word feedback (n = 92) and visual circle (n = 89). In the implicit condition (172 participants included), there were two between-subjects conditions, word feedback (n = 87) and visual circle (n = 85). In all conditions, feedback epochs were triggered based on the same criteria from earlier experiments, when there were three consecutive accurate responses on city trials where the mean RT for those three trials was 1.0 SD or greater below the mean RT up to that point in the experiment. These conditions were all randomly assigned to participants; all other aspects of the design were identical to Experiment 2.

### Results

#### Self-report

As in Experiments 1 and 2, we collected the same self-report measures. Analyses were conducted only on participants who answered each self-reported question dependent on that particular analysis.

A 2 × 2 between-subjects ANOVA of instruction type (explicit or implicit) and feedback epoch (word feedback vs visual circle) was run on each descriptive statistic. The groups did not show a difference in whether they thought the feedback epochs were affecting their performance, more or less annoying, or self-reported mind-wandering, all *p*s > 0.05. Similar to Experiment 1, on average participants regardless of condition did find the feedback epochs to be annoying (M: 3.58) more so than helpful (M: 3.43) or positive (M: 3.40), though these differences did not reach statistical significance in paired sample t-tests, *p*s > 0.05. See Table 3 for all means and test statistics.

**Table 3:** Statistic descriptives using t-test for equality of means

	Explicit Feedback	Explicit Circle	Implicit Feeback	Implicit Circle	*F*-statistic (Instruction, Feedback)
	Mean (SD)	Mean (SD)	Mean (SD)	Mean (SD)	
Helpful	3.53 (2.30)	3.23 (2.14)	3.57 (2.27)	3.31 (2.12)	0.01, 0.92
Changes	3.33 (2.06)	3.51 (2.09)	3.07 (1.97)	3.74 (2.07)	0.01, 3.58
Positive	3.32 (1.96)	3.35 (2.21)	3.47 (2.13)	3.47 (2.01)	0.32, 0.01
Annoying	3.40 (2.12)	3.44 (2.33)	3.32 (2.31)	4.18 (2.26)	1.78, 3.41
Mind-wandering	3.12 (1.65)	3.17 (1.64)	3.33 (1.68)	3.54 (1.59)	2.59, 0.54

*p* ’s > .05

#### Total feedback epochs

A 2 × 2 between-subjects ANOVA of instruction (explicit or implicit) and feedback epoch (word feedback vs visual circle) were run on total feedback epochs, all *ps* > 0.05. Overall, we did not find more feedback epochs occurred in the explicit group (M: 5.35) compared to the implicit group (M: 5.38). Additionally, we did not find more feedback epochs occurred in the word group (M: 5.22) compared to the circle group (M: 5.51). These data suggest that in a sustained attention task, the type of feedback triggered by periods of fast responding does not change how frequently participants enter those periods of fast responding.

#### Global analyses

We examined measures of RT, CV, commission errors, and correct commission rates across each block of trials, the same measures from Experiment 2. Responses on trials during which feedback was occurring were not included in these analyses. To be consistent with previous analyses, we also looked at each of these measures only within the final block of 100 trials in a separate analysis.

A 2 × 2 × 4 mixed factor ANOVA was run on RT, CV, commission errors, and correct commission rate with factors of block (1, 2, 3, or 4) instruction (explicit or implicit) and feedback epoch (word feedback vs visual circle). For RT, there was an interaction between instruction and feedback epoch *F*(1, 349) = 4.37, *p* = 0.037, η_p_^2^ = 0.01. Simple main effects analyses revealed that for word feedback, RTs were shorter in the implicit condition (699 ms) compared to the explicit condition (718 ms), *p* = 0.05, whereas there was no significant difference in the circle feedback condition, *p* = 0.33.

For CV, there was a significant effect of block *F*(3, 1047) = 190.41, *p* < 0.001, η_p_^2^ = 0.35, where CV increased as time on task increased. For commission errors, there was a significant effect of block, *F*(3, 1047) = 140.10, *p* < 0.001, η_p_^2^ = 0.29, where commission errors increased as time on task increased. These results replicate similar patterns to previous findings in Experiments 1 (and prior research, e.g., Rosenberg et al., 2013) in which errors and variance increase in the gradCPT as the task progresses. For correct commission rate, there again was a significant effect of block *F*(3, 1047) = 51.73, *p* < 0.001, η_p_^2^ = 0.13, where performance decreased as time-on-task increased. There were no other main effects or interactions found (*p*s > 0.05). See Table 3 for means and standard deviations.

Finally, we conducted 2 × 2 ANOVAs to compare commission errors, correct commission rate, CV, and RT for just the last 100 trials across the instructions and feedback epochs. There were no differences between either instruction nor feedback epochs for CV, commission errors, or correct commission rates. There was an interaction between instruction and feedback epochs for RT, *F*(1, 349) = 5.38, *p* = 0.02, η_p_^2^ = 0.02, reflecting a similar pattern to the interaction observed across all blocks, but no main effect of instruction or feedback epochs (*p*s > 0.05).

Apart from one relatively modest overall interaction for RT, there were no global differences in any of our measures across the various types of feedback and instruction. Overall, this indicates that the specific type of feedback, or whether the participant is aware that feedback is triggered by their behavior, has little effect on global task performance.

#### Local analyses

We conducted 2 × 2 × 2 × 2 mixed factorial ANOVAs with within-subjects factors of feedback type (visible vs. invisible) and time (before vs. after feedback epochs) and between-subjects factors of instruction (explicit vs. implicit) and feedback epoch (word feedback vs visual circle). These were conducted for all the same dependent variables as the prior experiments.

**Fig. 7:**
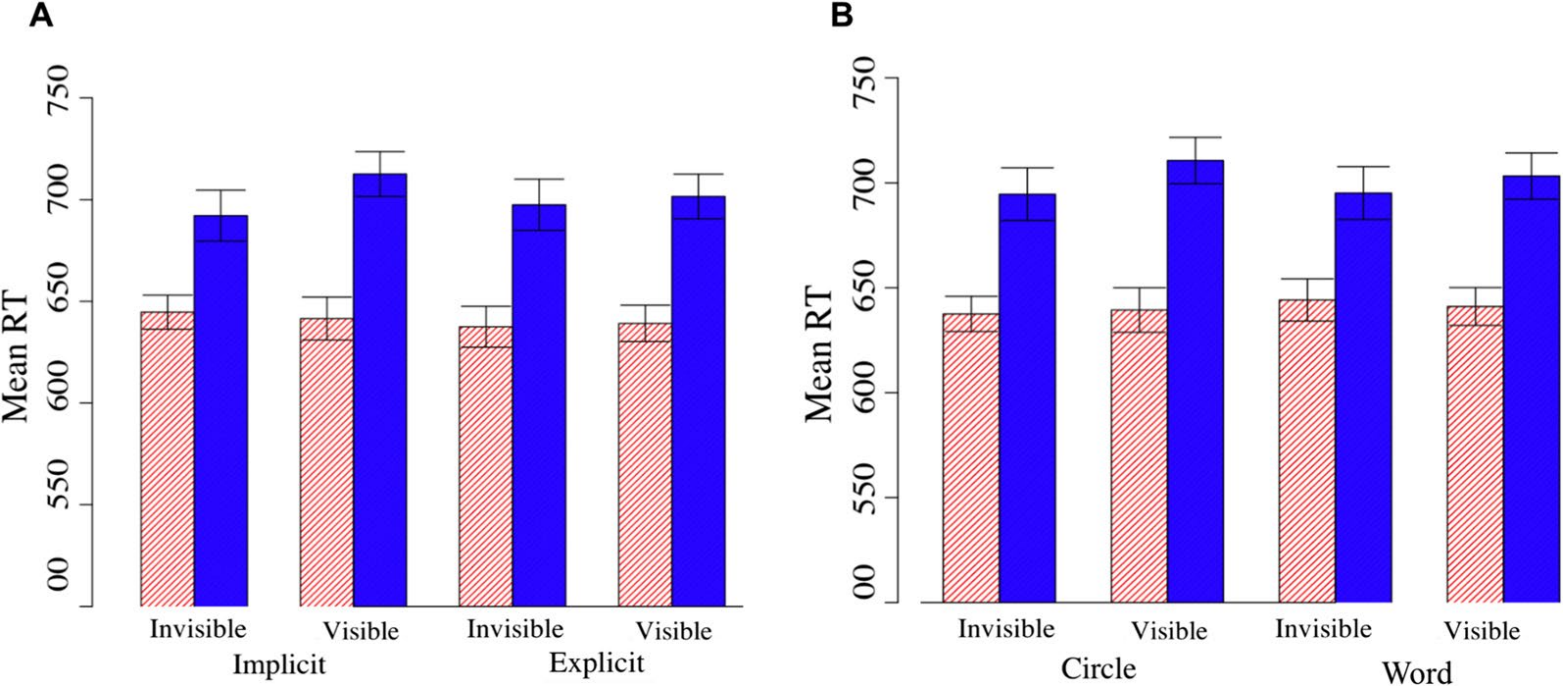
Mean RT of before and after visible and invisible feedback epochs. **A** for explicit vs implicit instructions (collapsed across feedback epochs) and **B** for word feedback vs visual circle feedback epochs (collapsed across instruction type)

There was a main effect of time on RT, *F*(1, 343) = 345.41, *p* < 0.001, η_p_^2^ = 0.50, such that before a feedback epoch, mean RT was 640 ms, and after a feedback epoch mean RT was 701 ms. This was expected, as the feedback epochs were triggered specifically by periods of atypically short RTs. Critically, there was one significant interaction for RT, between time and feedback type, *F*(1, 343) = 4.24, *p* < 0.01, η_p_^2^ = 0.01. Simple main effects analyses revealed that prior to the feedback, RT was not significantly different between the invisible (641 ms) and visible (640 ms) condition, *F*(1,343) = 0.059, *p* = 0.81. However, after the feedback, RTs were significantly longer in the visible condition (708 ms) compared to the invisible condition (695 ms), *F*(1, 343) = 4.66, *p* = 0.03 (Fig. 7). No other main effects or interactions were found (*p*s > 0.05).

This result is critical in two respects. First, it replicates our finding from Experiment 2, that feedback triggered by atypically fast periods of responding can in fact slow subsequent responses. Second, it demonstrates that this effect does not interact with the specific type of feedback (words or circles), or whether the participant has knowledge that the feedback is linked to performance (implicit vs. explicit). In other words, the slowing of responses following performance-triggered feedback is robust across a variety of contexts.

The same analysis with CV as a dependent variable exhibited no main effects or interactions, *p*s > 0.05. For correct commissions, there was a significant main effect of time, *F*(1, 343) = 38.66, *p* < 0.001, ηp^2^ = 0.11. Accuracy before a feedback epoch was 97.2% and after a feedback epoch was 94.6%. This was expected because in order to trigger an alert, participants needed to have three consecutive accurate and fast responses to city trials. There were no other significant main effects or interactions (*p*s > 0.05).

**Fig. 8:**
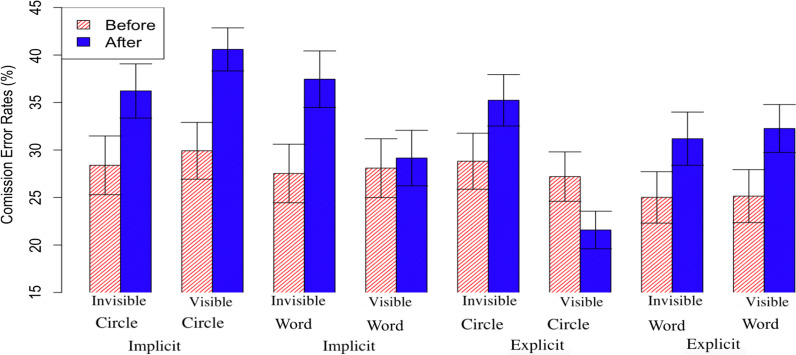
Mean commission error rates (%) of before and after visible and invisible feedback epochs for explicit vs implicit instructions and word feedback vs visual circle feedback epochs

Finally, for commission errors, there was a significant main effect of time, *F*(1, 253) = 8.63, *p* < 0.01, ηp^2^ = 0.03, with more errors after a feedback epoch (35.5%) compared to before a feedback epoch (28.0%). Notably, as in Experiment 2, there was no interaction between time and feedback type, *F*(1, 253) = 1.60, *p* = 0.21, ηp^2^ = 0.006. This suggests that the increase in errors is not due to the appearance of the feedback itself, but instead occurs regardless following a period of atypically fast responding. There was also a three way interaction between instruction, feedback epoch, and feedback type, *F*(1, 253) = 4.75, *p* = 0.03, ηp^2^ = 0.018. See Additional file 1: Table S3 and Fig. 8 for means of commission errors. Critically, there were no other main effects or interactions for any remaining measures, all *p*s > 0.05. In other words, all other measures were otherwise not differentially affected by whether the feedback was visible or invisible or if the participants were in the fast triggered or slow triggered condition.

**Fig. 9:**
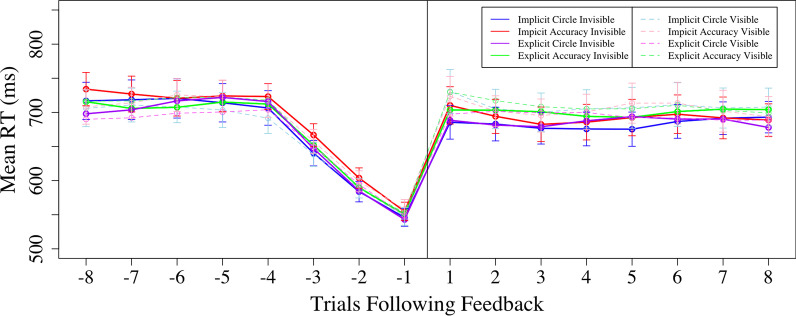
Time window of RT before and after feedback epochs for the implicit vs explicit instructions and word feedback vs visual circle conditions for visible and invisible feedback epochs. *Note:* The negative trials on the x-axis are trials before the feedback was given and the positive trials are the trials following feedback

As in Experiments 1 and 2, since the size of the time window was somewhat arbitrary, we conducted a follow-up analysis examining RT at a more fine-grained level (Fig. 9). Please refer to Experiment 1 to see how we created a moving window average. As can be seen in Fig. 9, there is a clear pattern in the triggered trials in which RT was decreasing leading up to the triggering of the feedback epochs, as would be expected based on the algorithm that was used to trigger those feedback epochs. As seen similarly to Experiment 2, following feedback, RTs are higher in the visible condition compared to the invisible condition.

We conducted a 2 × 2 × 2 × 2 × 8 ANOVA with within-subjects factors of feedback type, time, and trial, and between-subjects factors of instruction and feedback epoch. There were main effects of trial and time, *p*s < 0.001, both mediated by interactions. As in Experiment 2, there was a significant interaction between feedback type and time, *F*(1,2345) = 7.95, *p* = 0.005, ηp^2^ = 0.02. Simple main effects analyses revealed that RTs were longer following visible (705 ms) compared to invisible (691 ms) feedback, *F*(1,335) = 6.81, *p* = 01. However, prior to feedback epochs, there was no effect of feedback type, *F*(1,335) = 1.18, *p* = 28. These results replicate previous findings from Experiment 2 and reaffirm our analysis in the section above, emphasizing that visible performance-triggered feedback does increase RT.

There was also once again a significant interaction between time and trial, *F*(1,2345) = 322.22, *p* < 0.001, ηp^2^ = 0.49, reflecting a change in RTs over trial prior to the feedback epoch but not after. This was expected based on how feedback epochs were triggered and matches what was observed in prior experiments. No other main effects or interactions were significant, *p*s > 0.05.

## Discussion

The goal of the present study was to determine whether performance-linked feedback could improve performance in a sustained attention task. We found evidence that real-time visual feedback can modify participants’ performance. Specifically, short feedback epochs linked to participant performance, that were triggered when participants were responding significantly faster than usual, caused participants to subsequently slow their responses. In Experiment 1, we demonstrated that these longer RTs occurred only when feedback was linked to performance; when feedback occurred at predetermined intervals, feedback epochs did not produce longer RTs. In Experiment 2, we showed that the longer RTs produced by feedback epochs were not a result of a natural process that would occur with or without feedback; when feedback epochs were not visible after being triggered by a period of fast responding, the same return to baseline in RT did not occur. In Experiment 3, we found that the RT slowing effect was robust across different types of feedback as well as conditions in which participants explicitly knew about the link between their own performance and the feedback.

Prior studies have found that shorter RTs are linked to increased errors (e.g., Rosenberg et al., 2013). Thus in theory, this kind of performance-linked feedback could improve performance in sustained attention tasks by slowing participants down. However, we did not observe a reduction in errors immediately following feedback epochs. Instead, performance overall appeared to get worse following feedback epochs regardless of whether those feedback epochs were linked to performance. However, in Experiments 2 and 3, we noted that this was also the case following invisible feedback. Therefore, a plausible explanation is that following periods of atypically fast responding, participants are more prone to make mistakes regardless of whether feedback is presented. This is in line with previous research showing that errors are typically high when participants enter periods of atypically fast responding (e.g., Rosenberg et al., 2013). The feedback does not reduce those errors, but it also does not exacerbate this problem. More broadly, this suggests that while the feedback epochs implemented in the current study were successful in demonstrating an ability to manipulate performance in real-time based on performance-based feedback, they were not effective in improving performance overall. Perhaps this is linked to participants’ perception of this feedback, as participants did generally report finding the feedback to be annoying across the experiments.

The repeated RT slowing effect in all three experiments is promising in that real-time triggering of visual feedback serves as an effective intervention to detect and interrupt lapses of sustained attention and ultimately modify participants’ behavior. While we failed to reduce the rate of commission errors in the current study, there may be real-world applications where slowing by itself is a sufficiently useful outcome.

These results build on prior literature on cueing during sustained attention tasks. For example, rather than using a trailing mean like in the present study, Manly et al. (2004) established a mean RT threshold that was utilized to trigger non-specific, simple tone auditory cues whenever participants’ response times decreased and fell below the determined threshold. The mean RT was calculated from a modified SART that the participants completed prior to the experimental trials. The authors found that the cued condition significantly improved accuracy, though whether the cues were triggered by below threshold responses compared to presented at random intervals did not matter, it was the mere presence of cues in general that caused the effect. As noted by Manly et al. (2004), examining RT more closely after the auditory cue reveals a brief and short-lasting period of increased RT. We have reliably reproduced this RT slowing effect with visual feedback epochs in three experiments. There were several key differences between the two studies, however. First, in Manly et al., participants were explicitly instructed to use the cues to stay on task, whereas we gave no explicit instructions related to feedback epochs in the current study. Second, in their study, cues improved performance regardless of whether they were tied to performance or not. In our Experiments 2 and 3, we demonstrated that cues could change performance specifically when tied to a particular level of performance. Finally, their cues reduced errors whereas ours did not. This may be because of differences in modality, differences in instruction, differences in task, or other factors that may be examined in future research.

Future research might adapt this method to explore the near limitless number of possible ways to intervene at critical moments. There is still a great deal to learn about what interventions may be effective and how people respond to different types of warning systems. In earlier pilot testing, for example, we tested alerts that instructed participants to focus but found that participants were extremely annoyed at this intervention (in some cases even quitting the task in anger). Another issue with the current approach is that there are relatively few mountain trials, and thus the data on commission errors following feedback is noisy. For example, in Fig. 8, it appears that in the explicit condition with circle feedback, commission errors are actually reduced following feedback. However, the relevant interaction did not reach significance. In future research, there might be alternative methods that are more sensitive to commission errors following feedback, such as bursts of mountain trials or other approaches.

Additionally, Seli et al. (2012) investigated speed-accuracy tradeoffs in the SART. When task instructions emphasized accuracy, errors decreased. However, sustained attention task instructions typically prioritize both speed and accuracy. Therefore, participants may occasionally sacrifice accuracy in an effort to maintain consistent speed. Interestingly, however, specific auditory alerts that participants were instructed to use to increase awareness during the sustained attention task resulted in increased errors and RT variability. This result somewhat parallels the present findings. Visual feedback epochs of “Correct!” and “Incorrect”, or green and blue circles, simultaneously provided participants with information pertaining to their accuracy as well as alerting participants to potential mind-wandering. While providing feedback about accuracy can decrease errors in some circumstances, in other cases feedback may serve more as a distraction than anything else, increasing errors and RT variability. As such, some of the benefits the present study may have had with providing information about accuracy may have been negated by the distracting nature of the visual feedback epoch itself. Future research may explore other types of visual or non-visual feedback in the hopes of minimizing distraction while maximizing the benefits of interrupting performance when attention wanes.

Notably, another study utilized “speeding tickets” consisting of several irritating beeps and text on the screen to slow down participants that were responding too quickly during a visual search task (Wolfe et al., 2007). The speeding ticket was only triggered when an observer indicated a target was absent with an RT shorter than the determined speed limit for that particular type of trial. This approach considerably slowed down responses; similar to the present study, however, the sizable slowing effect caused by the speeding tickets did not significantly alter response accuracy. There were notable differences between this result and the present study; for example, no accuracy feedback was involved with speeding tickets, and observers who received a speeding ticket were also required to complete a second response following the speeding ticket trial. Still, these results are broadly consistent with our findings in that it is possible to slow participants down when they would otherwise be responding too quickly, but this does not mean that they will reduce their errors. We are hopeful that the current methodological approach provides a valuable new tool to study these possible interventions and test their efficacy when linked to real-time performance. Because we were able to test these interventions online, the current approach provides a template for flexible and rapid testing of different intervention approaches to improve performance in sustained attention tasks.

Supplementing the underload and overload theories of sustained attention, several neurocognitive models have been proposed. Esterman and Rothlein (2019) describe four such models: arousal, control, opportunity cost, and efficiency. In the arousal model, sustained attention is situated on a bell-curve of physiological arousal, reminiscent of the Yerkes Dodson law. Hypoarousal and hyperarousal in this model are correlated with low and high locus coeruleus activity, respectively. Research supporting the arousal model of sustained attention presupposes that mind-wandering/underload is responsible for generating lapses of sustained attention. The control model, based on the resource-control theory, dictates that different brain regions are engaged in a continuous battle for attentional resources. Generally, there is an inverse relationship between the default mode network (mind wandering) and the frontal-parietal and dorsal attentional networks (attentional control). However, Thomson et al. (2015) notes that attentional resources will always be biased towards mind-wandering. In the opportunity cost model, cognitive control is dependent on how the observer subjectively values the task compared to other mental activities. The opportunity cost model suggests that motivation to receive rewards can serve as a powerful modulator of task performance. Lastly, the efficiency model dictates that optimal visual processing occurs during “in-the-zone” periods when less computational resources and effort are required (Esterman & Rothlein, 2019). Together, these four neurocognitive models offer different approaches to the same goal of explaining how observers maintain sustained attention in a challenging visual task.

The present study utilized the deBettencourt et al. (2019) RT triggering paradigm that was rooted in the underload theory, such that the visual feedback epochs in the present study could be viewed as approaching reducing lapses in sustained attention from the perspective of mind-wandering. Conversely, if the overload theory is in fact the cause of vigilance decrements, then the additional stimulation involved in visual feedback epochs may contribute to further overload. In this context, the RT slowing effect may be due to the resource control theory (i.e. participants draining their attentional resources from overload rather than a purposeful pause following the visual feedback epoch). Future studies should elucidate the true cause of the RT slowing effect, specifically whether visual feedback epochs triggered during lapses of sustained attention cause participants to purposefully slow down or experience overload. For example, eye-tracking can be utilized to measure mind wandering with the gradCPT. Van den Brink et al. (2016) found that lapses in attention (commission errors) coincided with a smaller pupil diameter.

Future research could explore optimal interventions along a variety of dimensions including other modalities (e.g., auditory alerts), longer or shorter periods of feedback, or other types of performance-triggered interventions that may be effective in reducing errors (e.g., Hester et al., 2017; Lees et al., 2011; Nees et al., 2016) as well. For example, prior research has shown that an increase in CV may be a better predictor of lapses in sustained attention than faster or slower RT (e.g., Rosenberg et al., 2013). This suggests future research that focuses more on slower RT or CV might highlight other ways to track behavior and intervene at an optimal time. It is notable that attitudes about intervention may also be important. Our pilot data suggest that directly instructing observers to focus at points of inattention may elicit angry responses. However, we also observed in Experiment 3 that instructions at the beginning of the experiment that explicitly link the feedback to lapses in focus have little effect on overall performance. Thus, one challenge for future research is to find ways to intervene that change behavior without eliciting emotional responses that might counteract the benefits of the interventions.

In occupations such as radiology or baggage screening, where lapses of sustained attention can result in serious consequences such as missing the presence of a tumor or weapon, it is critical that we develop alert systems that can detect these periods of mind-wandering to reduce human errors. For example, radiologists may interpret up to 4% of their caseload of radiological scans incorrectly every day (e.g., Berlin, 2007). In one specific study, when radiologists examining abdominal computed tomography (CT) scans exceed a daily caseload of more than twenty radiologic examinations, error rates more than doubled (FitzGerald, 2005). While 4% may seem like a low number, any mistake could result in further harm to the patient or in the worst case scenario, death. In the case of airport baggage screening, the Transportation Security Administration discovered an average of 12.1 firearms in carry-on luggage per day in 2019. Out of the 4432 firearms that were confiscated that year, 87% were loaded (Wagner, 2020). If baggage screening agents were to miss any of these loaded firearms, they could endanger the lives of passengers and crew alike. Ideally, a system that could effectively monitor performance in real-time and trigger alerts during lapses of sustained attention may help save lives across a number of professional applications.

Just like the passenger who blurts out “Watch out!” when the driver approaches a roadside hazard, the visual feedback alert system can slow you down, but may not always prevent you from making a mistake. However, the fact that visual feedback repeatedly demonstrates a slowing effect across three experiments gives us confidence that the performance triggered interventions are capable of modulating behavior during sustained attention tasks without requiring the participant to stop performing the task. We are hopeful that the current studies provide a blueprint for future work that can explore the implementation of interventions that can not only slow people down but also prevent them from making mistakes. Developing a real-time intervention system that can do both is of utmost importance to preventing life-threatening mistakes that occur when sustained attention lapses across a number of real-world domains.

### Supplementary Information

**Additional file 1.** Statistic descriptives of means and standard deviations.

## Data Availability

All relevant subject-level data are publicly available at https://osf.io/mkgej/. Raw data and other materials are available from the authors on reasonable request.

## Appendix A

1. On a scale from 1 to 7 (1 = not at all, 7 = very much), please indicate which best represents the degree to which you felt your mind wandered during the task you just completed. I noticed visual feedback occasionally during the experiment (not just the practice)—if you answered no (note: this does not occur in some conditions, so if you didn't see it, that doesn't necessarily mean anything went wrong on your end), you can skip the next four questions. (yes/no question)
2. On a scale from 1 to 7 (1 = not at all, 7 = very much), did you find the visual feedback during the experiment to be helpful?
3. On a scale from 1 to 7 (1 = not at all, 7 = very much), did you feel that the visual feedback during the experiment was responding to changes in your performance (that it turned on because of something you did)?
4. On a scale from 1 to 7 (1 = not at all, 7 = very much), do you think the visual feedback during the experiment positively affected your ability to focus on the task?
5. On a scale from 1 to 7 (1 = not at all, 7 = very much), did you find the visual feedback during the experiment to be annoying?
6. Any other thoughts or comments? (optional)

## Abbreviations

RT: Response time
CPT: Continuous performance task
gradCPT: Gradual-onset continuous performance task
fMRI: Functional magnetic resonance imaging
rtfMRI: Real-time fMRI
ASRS-v1.1: Adult ADHD Self-Report Scale
RTSD: Response time standard deviation
CV: Coefficient of Variation
CT: Computed tomography

## Footnotes

1 We also provide measures of response time standard deviation (RTSD) on our data files at our OSF website, https://osf.io/mkgej/.

2 We thank an anonymous reviewer for raising these points.
