# Supplementary material for: Performance-linked visual feedback slows response times during a sustained attention task
Source: Cogn Res Princ Implic. 2023 May 29;8:32. doi: 10.1186/s41235-023-00487-w (PMC10226944; doi:10.1186/s41235-023-00487-w)
Supplement: Supplementary file 1 — Additional file 1. Statistic descriptives of means and standard deviations. [file 41235_2023_487_MOESM1_ESM.docx]

**Additional file 1**

*Table S1:* Statistic descriptives of means and standard deviations

|  | Predetermined |  |  |  |
| --- | --- | --- | --- | --- |
| Block | 1 | 2 | 3 | 4 |
|  | Mean (SD) | Mean (SD) | Mean (SD) | Mean (SD) |
| RT (ms) | 693 (68.23) | 686 (81.06) | 681 (83.73) | 690 (79.03) |
| CV (ms) | 16 (5.70) | 20 (7.43) | 21 (7.58) | 23 (8.45) |
| Commission Errors (%) | 20.75 (13.40) | 31.27 (16.51) | 34.31 (18.36) | 33.76 (17.74) |
| Correct Commissions (%) | 97.78 (4.34) | 95.32 (9.35) | 95.11 (10.77) | 92.87 (11.84) |
|  | Triggered |  |  |  |
| Block | 1 | 2 | 3 | 4 |
|  | Mean (SD) | Mean (SD) | Mean (SD) | Mean (SD) |
| RT (ms) | 686 (50.34) | 679 (66.30) | 676 (63.47) | 682 (67.83) |
| CV (ms) | 15 (6.01) | 20 (7.87) | 21 (7.50) | 22 (8.43) |
| Commission Errors (%) | 21.48 (14.83) | 31.90 (15.94) | 32.05 (17.06) | 33.13 (19.37) |
| Correct Commissions (%) | 98.42 (4.39) | 96.83 (5.55) | 96.72 (6.09) | 95.22 (9.50) |

*Table S2:*Statistic descriptives of means and standard deviations

|  | Explicit Circle |  |  |  |
| --- | --- | --- | --- | --- |
| Block | 1 | 2 | 3 | 4 |
|  | Mean (SD) | Mean (SD) | Mean (SD) | Mean (SD) |
| RT (ms) | 714 (57.55) | 708 (61.73) | 711 (66.15) | 716 (94.46) |
| CV (ms) | 17 (5.51) | 22 (6.29) | 21 (7.09) | 22 (6.59) |
| Commission Errors (%) | 19.04 (16.54) | 33.39 (16.52) | 33.60 (20.73) | 38.19 (21.77) |
| Correct Commissions (%) | 95.99 (6.12) | 91.73 (11.09) | 92.27 (12.22) | 89.94 (15.75) |
|  | Explicit Feedback |  |  |  |
| Block | 1 | 2 | 3 | 4 |
|  | Mean (SD) | Mean (SD) | Mean (SD) | Mean (SD) |
| RT (ms) | 701 (68.53) | 697 (62.90) | 697 (72.48) | 703 (74.07) |
| CV (ms) | 16 (5.20) | 22 (7.53) | 21 (8.27) | 23 (7.66) |
| Commission Errors (%) | 20.82 (17.51) | 34.93 (16.54) | 36.46 (19.40) | 36.95 (19.61) |
| Correct Commission (%) | 97.77 (3.87) | 94.79 (7.68) | 95.11 (9.33) | 93.97 (9.52) |

|  | Implicit Circle |  |  |  |
| --- | --- | --- | --- | --- |
| Block | 1 | 2 | 3 | 4 |
|  | Mean (SD) | Mean (SD) | Mean (SD) | Mean (SD) |
| RT (ms) | 705 (64.36) | 701 (64.41) | 709 (70.29) | 698 (67.11) |
| CV (ms) | 17 (5.74) | 22 (5.94) | 21 (6.78) | 22 (6.69) |
| Commission Errors (%) | 20.36 (16.14) | 34.79 (15.78) | 34.62 (18.79) | 35.80 (19.53) |
| Correct Commission (%) | 97.07 (4.50) | 93.82 (8.22) | 93.97 (9.37) | 92.87 (10.79) |

|  | Implicit Feedback |  |  |  |
| --- | --- | --- | --- | --- |
| Block | 1 | 2 | 3 | 4 |
|  | Mean (SD) | Mean (SD) | Mean (SD) | Mean (SD) |
| RT (ms) | 716 (65.63) | 715 (65.51) | 716 (74.30) | 723 (69.18) |
| CV (ms) | 16 (4.69) | 21 (6.02) | 21 (6.54) | 22 (7.08) |
| Commission Errors (%) | 19.14 (17.81) | 30.80 (14.83) | 28.45 (17.61) | 32.84 (18.63) |
| Correct Commission (%) | 97.48 (4.58) | 93.58 (7.44) | 93.54 (9.26) | 93.23 (9.21) |

*Table S3:* Statistic descriptives of means and standard deviations of commission errors

|  | Comission Errors % |  | Comission Errors % |
| --- | --- | --- | --- |
| Invisible |  | Visible |  |
|  | Mean (SD) |  | Mean (SD) |
| Implicit Words | 35.47 (34.84) | Implicit Words | 28.06 (30.14) |
| Implicit Circle | 32.23 (33.71) | Implicit Circle | 37.73 (32.16) |
| Explicit Words | 30.28 (34.07) | Explicit Words | 30.29 (32.73) |
| Explicit Circle | 32.08 (33.97) | Explicit Circle | 29.00 (32.86) |
